# Combination of transcriptome sequencing and iTRAQ proteome reveals the molecular mechanisms determining petal shape in herbaceous peony (*Paeonia lactiflora* Pall.)

**DOI:** 10.1042/BSR20181485

**Published:** 2018-12-11

**Authors:** Yanqing Wu, Yuhan Tang, Yu Jiang, Daqiu Zhao, Jiali Shang, Jun Tao

**Affiliations:** 1 College of Animal Science and Technology, Yangzhou University, Yangzhou 225009, P.R. China; 2 Key Laboratory of Crop Genetics and Physiology of Jiangsu Province, College of Horticulture and Plant Protection, Yangzhou University, Yangzhou 225009, P.R. China; 3 Ottawa Research and Development Centre, Science and Technology Branch, Agriculture and Agri-Food Canada, Canada

**Keywords:** APETALA2, P. lactiflora, proteome, transcriptome

## Abstract

The molecular mechanisms controlling petal shape in a herbaceous peony, *Paeonia lactiflora* Pall., a popular high-grade cut flower worldwide, remain unclear. Here, we selected inner and outer petals from *P. lactiflora* ‘ZiFengyu’ with an anemone type as the study object. Using transcriptome sequencing and isobaric tags for relative and absolute quantitation proteome, 979 differentially expressed genes and 266 differentially expressed proteins were detected within the inner and outer petals. Of these, the present study identified a key gene *APETALA2* that regulates flower shape development. In addition, we obtained a 1935 bp full-length cDNA sequence of *APETALA2* by rapid amplification of cDNA ends amplification. Through further validation using quantitative real-time polymerase chain reaction and Western blot analysis, *APETALA2* showed a markedly higher expression in outer than that in inner petals. Therefore, the present study indicates that the increased expression of *APETALA2* contributes to the formation of petals in *P. lactiflora*.

## Introduction

*Paeonia lactiflora* Pall., a flowering plant, is native to China. This traditional floral symbol of China is renowned as the king of flowers together with other peony species. Flower color is an important determinant for ornamental value, and the flower organ is an ideal model system for studying the relationship between plant development, genetics, and evolution. The flower color and organ development of *P. lactiflora* have become important research topics. Currently, the molecular mechanism involved in the floral formation has been well studied in *P. lactiflora*. Zhao et al. [1] have systematically analyzed the molecular mechanism of color formation in *P. lactiflora* with red outer petals and yellow inner petals using RNA sequencing technology, and identified potential candidate genes that could be involved in the flavonoid metabolic pathway including phenylalanine ammonia-lyase (*PAL*), flavanone-3-hydroxylase (*F3H*), flavonol synthase (*FLS*), and anthocyanidin synthase (*ANS*). The petal pattern or flower shape is one of the important indicators for ornamental merit; therefore, an increasing number of studies have focused on the petal formation pattern of ornamental plants. The flower shapes of *P. lactiflora* are directly related to its ornamental and commercial merit. The molecular mechanisms underlying the regulation of flower shape have been mainly studied using model plants including *Arabidopsis* and *petunia* [2–6], suggesting the molecular models known as ABCDE models regulate flower development. However, the molecular mechanism underlying petal development of *P. lactiflora* remains unclear. Identification of potential genes that mediate petal formation is urgently needed to provide a theoretical guideline for the development of *P. lactiflora* varieties with diverse floral shapes.

At present, high-throughput -omics technologies including RNA sequencing (RNA-seq) transcriptomes and proteomics provide new approaches to explore and identify functional genes, proteins, and regulatory pathways that affect the growth and development of ornamental plants [7,8]. Therefore, the objective of the present study was to explore the molecular regulatory mechanism of the formation of petals in *P. lactiflora* by omics analysis. In the present study, due to the lack of published genomic data for *P. lactiflora*, the inner and outer petals of the anemone-type cultivar ‘ZiFengyu’ were used for the non-parametric transcriptome and the isobaric tags for relative and absolute quantitation (iTRAQ) proteome were used to identify differentially expressed unigenes and proteins. *De novo* sequencing and proteomic analysis were used for functional annotations to identify important functional genes and proteins that regulate floral organ development; meanwhile, quantitative real-time polymerase chain reaction (qRT-PCR) and Western blot analysis were used to verify differentially expressed of the potential genes and proteins between the inner and outer petal tissues. Our study revealed for the first time the detailed molecular mechanism of petal regulation in *P. lactiflora* using genomics and proteomics. The results of the present study provide a theoretical reference for the future molecular breeding of *P. lactiflora*, which has important theoretical value and application significance for the improvement of flower shapes and for promoting the sustainable development of the *P. lactiflora* industry.

## Materials and methods

### Experimental materials

**Figure 1:**
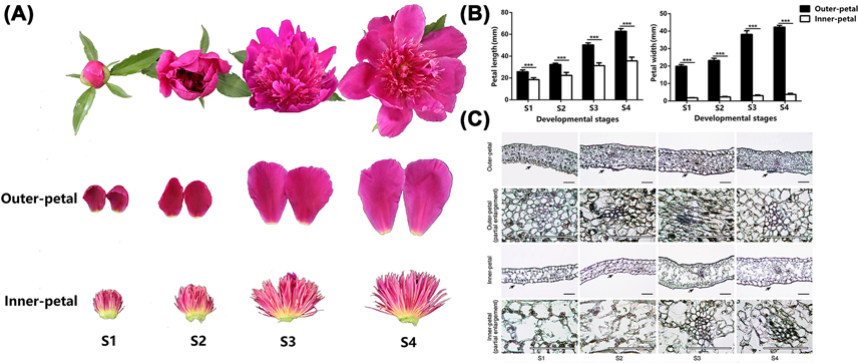
Petal shape characteristics of *P. lactiflora* ‘ZiFengyu’ during four developmental stages (S1, S2, S3, and S4) (**A**) Phenotypical, (**B**) morphological, (**C**) microstructural observations of outer and inner petals in *P. lactiflora* ‘ZiFengyu’ during four developmental stages. Black arrows in the first and third lines in the micrographs mark partially enlarged regions in the second and fourth lines; bars, 100 µm.

Experimental treatments were performed in the field of the germplasm repository of the Horticulture and Plant Protection College, Yangzhou University, Jiangsu Province, China (32°23′N, 119°24′E). *P. lactiflora* cultivar ‘ZiFengyu’ planted in 80 rows (two plants per row) was used as the experimental material. The plants grew well in field conditions with sufficient light and water supply. The flowers of each group had four developmental stages: Stage 1 (S1), flower-bud stage; Stage 2 (S2), initiating bloom stage; Stage 3 (S3), bloom stage; Stage 4 (S4), decline stage; flowers were collected from May 8 to May 20, 2016, respectively. Outer and inner petals were separated from the flowers as separate samples after each collection (Figure 1A). Subsequently, ten random outer and inner petals were selected from each group of the four developmental stages to measure the morphological indices—the length and width of outer and inner petals were measured by an electronic Vernier caliper (Guilin Guanglu Digitization Measuring Tools Co., Ltd., Guilin, Guangxi, China). Several samples were fixed with formalin-aceto-alcohol solution (45% (v/v) ethanol, 5% (v/v) acetic acid, and 5% (v/v) formaldehyde; FAA) for microstructure observation, and others were immediately frozen in liquid nitrogen, and then stored at −80°C until further analysis.

### Microstructure observation

The samples fixed in FAA solution were first dehydrated gradually in 50%, 70%, 90%, and 100% ethanol solutions and then dehydrated with a xylene and ethanol mixture (1/3, 1/1, 3/1; v/v) for 30 min each. Next, the samples were dehydrated twice with a xylene and chloroform mixture (9/1, v/v) for 30 min. After infiltration and embedding with paraffin, 8 μm sections were obtained using a rotary microtome (Model RM2245, Leica, Bannockburn, IL, U.S.A.). Additionally, after paraffin was removed using xylene, the sections were subsequently rehydrated in 100%, 95%, 80%, 50%, and 30% ethanol followed by distilled water. After finally being air dried, the sections were observed under a light microscope (Olympus CX31RTSF, Tokyo, Japan).

### Transcriptome sequencing and data analysis

Total RNA extracted from petals at S3 using a MiniBEST Plant RNA Extraction Kit (TaKaRa, Japan) were used for transcriptome sequencing. Six libraries (three outer petals and three inner petals) were prepared and sequenced by BGI Co., Ltd. (Shenzhen, China) using an Illumina HiSeq™ 4000 system (Illumina Inc., San Diego, CA, U.S.A.). After filtering of raw reads, transcriptome *de novo* assembly was performed using the Trinity short reads assembling program. The resulting sequences of Trinity were called Unigenes. Unigene annotation was performed using various bioinformatic databases, including the non-redundant protein database (NR), non-redundant nucleotide database (NT), gene ontology database (GO), cluster of orthologous groups of proteins database (COG), Kyoto encyclopaedia of genes and genomes database (KEGG), Swiss-Prot protein database (Swiss-Prot) and Interpro. The unigene expression level was calculated using the fragments per kilo bases per million reads method [9]. The threshold for significantly DEGs was set at a |log2(FoldChange)| > 1 and Probability ≥ 0.8. DEG functions were explored through GO and KEGG pathway analyses and the terms with *P*-value ≤ 0.05 were defined as significantly enriched. This was performed to identify significantly enriched metabolic pathways.

### iTRAQ proteome and data analysis

Petals samples for RNA-seq were also used to perform proteome analysis. Protein extraction was performed exactly as described previously by Zhao et al. [10]. After trypsin digestion, peptides were desalted with a C18 column (Phenomenex, Torrance, CA, U.S.A.) and dried in a spin vacuum. The desalted peptides (100 μg) were labeled with iTRAQ 6-plex reagent in 200 mM tetraethyl ammonium bromide according to the manufacturer’s instructions. Labeling was as follows: Inner-1, 124; Inner-2, 127; Inner-3, 128; Outer-1, 129; Outer-2, 130; Outer-3, 131. The labeled peptides with different reagents were combined, vacuum-dried, and separated on an LC-20AB high performance liquid chromatography pump system (Shimadzu, Japan) coupled with a high pH reversed phase column. Subsequently, the peptides were subjected to tandem mass spectrometry Q EXACTIVE (Thermo Fisher Scientific, San Jose, CA, U.S.A.) for data-dependent acquisition detection by nano-electrospray ionization.

Scaffold Q+ (version Scaffold_4.7.1, Proteome Software Inc., Portland, OR, U.S.A.) was used to quantitate TMT Label Based Quantification peptide and protein identifications. Normalization was performed iteratively (across samples and spectra) on intensities, as described in Statistical Analysis of Relative Labeled Mass Spectrometry Data from Complex Samples Using ANOVA (analysis of variance, Oberg et al. [11]). Partial least squares discriminant analysis (PLS-DA): PLS discriminant analysis is a type of classical partial least squares (PLS) regression (with a regression mode), which is used for classification and discrimination problems. The related analysis was completed by using the mixOmics package (https://CRAN.R-project.org/package = mixOmics). Volcano plot: The volcano plot, which plots significance versus fold-change on the *y* and *x* axes, respectively, is a type of scatter-plot that is used to quickly identify changes in large datasets composed of replicate data. It is drawn by using the ggplot2 package (http://ggplot2.org). GO analysis: Blast2GO version 4 was used for functional annotation. The whole protein sequence database was analyzed by BlastP using the whole NCBInr database; then this was mapped and annotated with a gene ontology database. Statistically, altered functions of differentially expressed proteins were calculated by Fisher’s exact test in BLAST2GO [12]. KEGG analysis: pathway analysis was processed by KOBAS (http://kobas.cbi.pku.edu.cn/). Pathways with a *P*-value <0.05 were recognized as significantly changed.

The total RNA of the *P. lactiflora* petal samples was extracted using a TaKaRa MiniBEST Plant RNA Extraction Kit (Bao Bioengineering (Dalian) Co., Ltd., Dalian, China) according to the manufacturer’s instructions. The RNA was then dissolved in diethylpyrocarbonate water and stored at −80°C for future use. About 1.0 μg of total RNA was used as a template for 3′ Rapid Amplification of cDNA Ends (RACE) and 5′ RACE amplification, respectively. 3′ RACE: The first strand of cDNA was synthesized using the 3′ and 5′-full RACE Core Set Kit (TaKaRa), and finished with two cycles of PCR according to the manufacturer’s instructions. The final PCR reaction solution was electrophoresed on a 1% agarose gel for purification. The target PCR fragments were recovered and ligated to the cloning vector pMD18-T for transformation, from which the transformed clones were selected for sequencing in Shanghai Bioengineering Co., Ltd., (Shanghai, China)

### qRT-PCR analysis

cDNA was synthesized from RNA using PrimeScript RT reagent Kit with gDNA Eraser (TaKaRa) and quantitated with an Eppendorf spectrophotometer (Eppendorf, Germany) at 260 nm. *P. lactiflora β-actin* (JN105299) was used as internal control. Q-PCR primer was designed using the Primer 5.0 program (PREMIER Biosoft International, Canada). For *APETALA2* (*AP2*) gene, forward primer: AGGAGGAAGAGGCAGAACCAAG, reverse primer: ATACACCACCACCAGCAGACC; For *β-actin* gene, forward primer: ACTGCTGAACGGGAAATT, reverse primer: ATGGCTGGAACAGGACTT. Real-time PCR amplification was performed in 25 μl reaction mixtures contained 12.5 µl 2× SYBR Premix Ex TaqTM, 2 µl cDNA solution, 2 µl mix solution of target gene primers and 8.5 µl ddH_2_O. PCR reactions were performed on an Applied Biosystems ABI 7500 Real-time PCR System. PCR cycling parameters were: 50°C for 2 min, followed by an initial denaturation step at 95°C for 5 min, 40 cycles at 95°C for 15 s, 51°C for 15 s, and 72°C for 40 s. The relative gene expression levels of target genes were calculated using the 2^−ΔΔ*C*^_t_ comparative threshold cycle (*C*_t_) method [13]. Each sample was tested three times to obtain the sample mean.

### Western blot analysis

Total protein was extracted using Trizol and analyzed using the bicinchoninic acid assay kit (Thermo Fisher Scientific Inc., Waltham, MA, U.S.A.). SDS-PAGE (polyacrylamide electrophoresis) of the protein samples (10 µl) was performed at 120 V for 90 min in a 10% gel. Each protein sample was transferred to a polypropylene difluoroethylene membrane and immunoblotted with the relevant antibody. Blocking solution and anti-AP2 (1:1000) synthesized by Abmart Co., Ltd. (Shanghai, China) was added at approximately 0.1 ml/cm^2^. Goat-Anti-Rabbit IgG-HRP (1:10,000) was used as a secondary antibody, and β-actin protein was used as a reference.

## Results and analysis

### Petal shape characteristics of *P. lactiflora* ‘ZiFengyu’ during four developmental stages

The length and width of the inner and outer petals were measured to provide a more accurate description of the changes in their shape. These measurements showed significant differences in each developmental period (*P*<0.05; Figure 1B). Particularly, mean width of the outer petals was approximately 11.13 times that of the inner petals. The lengths and widths of both the inner and outer petals increased with the progression of developmental stages. Compared with S1, the length and width of the outer petals in S4 had increased by 2.44 and 2.23 times, respectively, whereas the length and width of the inner petals had increased by 1.93 and 2.07 times, respectively. In addition, the microstructure of the inner and outer petals was observed using paraffin sections. The epidermis, palisade tissue, and vascular bundles were formed in the early developmental stage of the inner and outer petals in cultivar ‘ZiFengyu’ (Figure 1C). Microstructural comparison showed that palisade tissue in the outer petals was significantly tighter than in the inner petals.

### Transcriptome analysis

**Figure 2:**
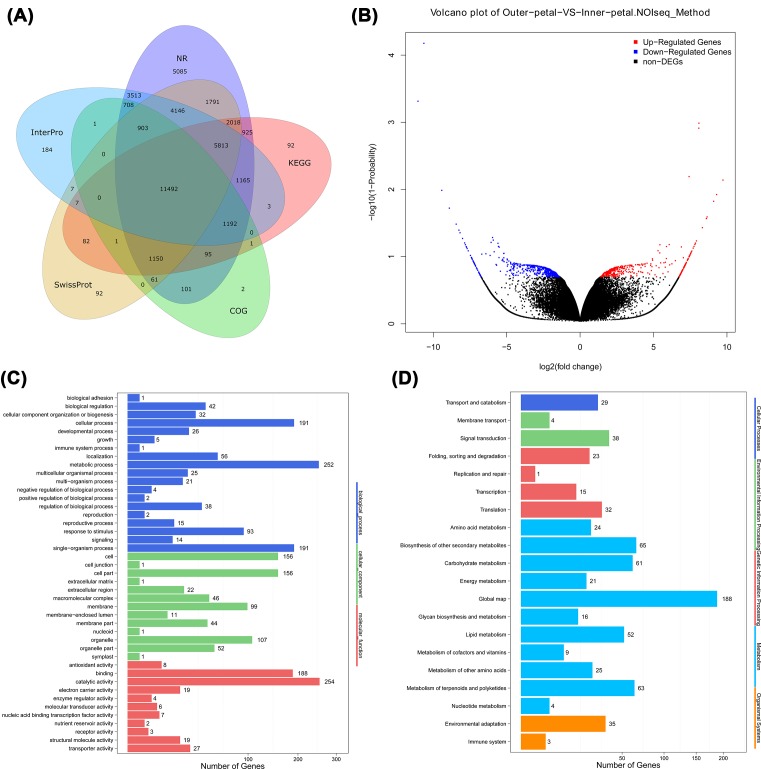
Transcriptome analysis of outer and inner petals in *P. lactiflora* ‘ZiFengyu’ (**A**) Functional annotation of all unigenes based on the following databases: non-redundant protein database (NR), cluster of orthologous groups of proteins database (COG), Kyoto encyclopaedia of genes and genomes database (KEGG), Swiss-Prot protein database (Swiss-Prot) and Interpro; (**B**) Volcano plot of DEGs. The *x* and *y* axes represents log2 fold change and the −log10(1 – Probablity) value, respectively. The red, blue, and black points represent up-regulated, down-regulated, and non-DEGs, respectively. (**C**) GO and (**D**) pathway classifications of DEGs where *x* axis represents the number of DEGs; the *y* axis represents (C) the GO terms or (D) the pathway terms.

To investigate the mechanism underlying the formation of *P. lactiflora* petals, we initially performed a comparative transcriptome between outer and inner petals using RNA-seq. Six cDNA libraries were constructed from outer petals (*n*=3) and inner petals (*n*=3). After quality assessment and data filtering, a total of 39.42 GB of clean data were obtained (NCBI accessions: SRP151426); the average clean reads ratios of outer and inner petals were 99.85% and 99.84%, respectively (Supplementary Table S1). The clean reads were assembled into a total of 66,293 high-quality unigenes with an average length of 1152 nt (Supplementary Table S2). Subsequently, these unigenes were annotated using seven public databases. In total, 66,293 unigenes were annotated, accounting for 65.93% of all unigenes. Among them, 40,158; 36,262; 27,563; 24,036; 15,707; 29,134; and 18,201 unigenes could be annotated to the NR, NT, Swiss-Prot, KEGG, COG, Interpro, and GO databases, respectively, accounting for 60.58%, 54.70%, 41.58%, 36.26%, 23.69%, 43.95%, and 27.46% of all unigenes, respectively (Figure 2A).

After comparative analysis, a total of 979 DEGs were obtained, including 408 up-regulated and 571 down-regulated DEGs (Figure 2B and Supplementary Table S3). To elucidate the functions of these DEGs, they were assigned to GO categories and grouped into the following three main categories: biological process, cellular component, and molecular function. Within the biological process category, 252, 191, and 191 DEGs were enriched in metabolic, cellular, and single-organism processes, respectively. In the cellular component category, cell (156 DEGs) and cell part (156 DEGs) were dominant functions. In the molecular function category, binding (188 DEGs) and catalytic activity (254 DEGs) accounted for a major proportion (Figure 2C). KEGG enrichment analysis of the DEGs was performed, and the top 20 KEGG pathways of DEGs are shown in Figure 2D; detailed data are presented in Supplementary Table S4. As a result, DEGs were mainly enriched in a global map, biosynthesis of other secondary metabolites, metabolism of terpenoids and polyketides, and in carbohydrate metabolism pathways.

### iTRAQ proteome analysis

**Figure 3:**
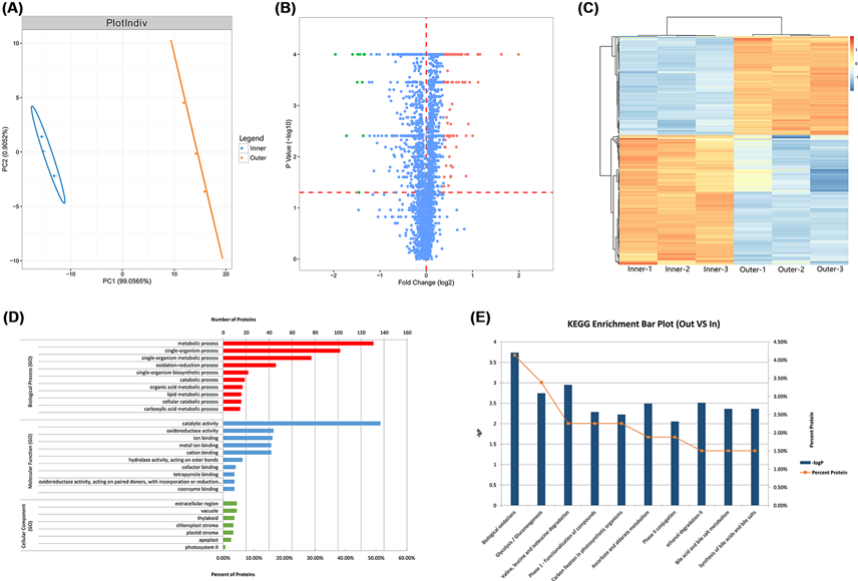
Isobaric tags for relative and absolute quantitation (iTRAQ) proteome analysis of outer and inner petals in *P. lactiflora* ‘ZiFengyu’ (**A**) Partial least squares discriminant analysis (PLS-DA) of petals samples; (**B**) Volcano plot of DEPs. The *x* axis represents log2 fold change; the *y* axis represents −log10(*P*-value); (**C**) Heat map clustering analysis of DEPs between outer and inner petals; (**D**) GO classification of DEPs; (**E**) Pathway classification of DEPs.

Proteome analysis of the same samples assayed by RNA-seq was performed using the iTRAQ method. PLS-DA analysis showed that the similarity of the three biological replicates within each group was sufficiently high (Figure 3A). A total of 266 differentially expressed proteins (DEPs) between outer and inner petals were obtained in *P. lactiflora* (Figure 3B and Supplementary Table S5), and heat map clustering analysis showed the expression pattern of DEPs (Figure 3C). GO function analysis showed (Figure 3D) within the biological process category that 131 and 102 DEPs were enriched in metabolic and single-organism processes, respectively; in cellular component, extracellular region (12 DEPs) and vacuole (12 DEPs) parts were dominant functions; in molecular function, catalytic activity (137 DEPs) accounted for a major proportion. KEGG enrichment analysis of the DEPs was performed, and the top 10 KEGG pathways of DEPs are shown in Figure 3E while detailed data are presented in Supplementary Table S6.

### Identification of key gene/protein regulating petal formation and expression validation

**Figure 4:**
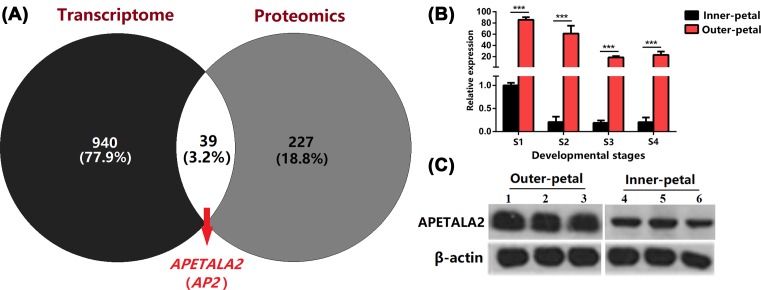
Differentially expressed validation of *APETALA2* between outer and inner petals in *P. lactiflora* ‘ZiFengyu’ (**A**) Venny analysis of transcriptome and proteome. (**B** and **C**) *APETALA2* expression detection of outer and inner petals in *P. lactiflora* ‘Zi Fengyu’ during four developmental stages (S1, S2, S3, and S4) using (B) quantitative real-time polymerase chain reaction (qRT-PCR) and (**C**) Western blot, lanes 1−3 represent three duplicated samples from outer-petals, lanes 4−6 represent three duplicated samples from inner-petals.

To identify the most important molecular regulation of flower shape, we performed comparative transcriptome and proteome analyses between outer and inner petals in *P. lactiflora*. Through the comprehensive analysis of transcriptome and proteome, we obtained 39 same differentially expressed genes/proteins, including important gene/proteins such as *APETALA2* (gene symbol Unigene11105_All) that are probably related to the formation of petals (Figure 4A). RACE amplification showed that the full-length cDNA sequence of *APETALA2* was 1935 bp, containing a 1578 bp open reading frame (ORF), 222 bp 5′-untranslated regions (5′-UTR), and 135 bp 3′-untranslated regions (3′-UTR) (Supplementary Figures S1 and S2). To validate the function, the expression levels of APETALA2 were analyzed by qRT-PCR (Figure 4B) and Western blot (Figure 4C). *APETALA2* showed a markedly higher expression in outer petals than that in inner petals.

## Discussion

Flower occurrence pattern, developmental processes, and structure have strong genetic stability, and are the main basis for the evolution and development of the angiosperm system, which are also important features that classical taxonomists give close attention. Therefore, flower organs are ideal model systems for studying the relationships among development, genes, and evolution [14]. The flower development governed by genes is an important stage of change in the process of plant growth and development, and also a highly complex period of physiological, biochemical, and morphological changes. At present, the molecular mechanism of floral organ development has mainly been studied in model plants including *Arabidopsis thaliana, Antirrhinum majus* and *Petunia hybrid*. Coen and Meyerowitz [15] proposed an ABC model for flower organ development. Angenent et al. [16] identified two genes *FLORAL BINDING PROTEIN 7* (*FBP7*) and *FLORAL BINDING PROTEIN 11* (*FBP11*) that governed ovule development in the model plant *Petunia* hybrid. Colombo et al. [5] proposed an ABCD model of flower organ development, followed by Pelaz et al. who identified gene *SEPALLATA1/2/3* (*SEP1/2/3*) in *Arabidopsis thaliana*. When *SEP1/2/3* was mutated at the same time, each flower organ developed into a sepaloid-like structure [2]. Therefore, Pelaz et al. [3] proposed an ABCDE model of floral organ development. In the present study, differentially expressed genes and proteins in the development of *P. lactiflora* flower organs were screened using transcriptomes and proteomics for the first time, and a key functional gene *APETALA2* (*AP2*) was identified. Gene *AP2*, as an A-class gene in the ABCDE model of flower organ development and regulation in plants, is involved not only in the establishment of floral meristems but also in the formation of sepals and petals [17]. Jofuku et al. [4] isolated the first *AP2* gene from the model plant *Arabidopsis thaliana*, and then from maize (*Zea mays*), peony (*Paeonia suffruticosa*), apple (*Malus domestica*), and strawberry (*Fragaria ananassa*) [18–21]. In the present study, according to the transcriptomic sequencing, the full-length cDNA (1935 bp) of the *APETALA2* gene was successfully cloned with RACE, which provides favorable fundamental knowledge for further study of *APETALA2* gene function by qPCR and overexpression techniques.

The *APETALA2* gene is an important factor in the establishment of the floral meristem. It is different from other genes of the MADS-box family, and it participated in plant growth and development as well as in various physiological and biochemical reactions. The *APETALA2* gene, which is known to be the A-class gene in flower organs, encodes a family of AP2/EREBP transcription factors and plays a central role in the establishment of floral meristems as well as in the formation of sepals and petals [17,22]. Previous study in water lily revealed the expression level of *NsAP2* gene could be ranked as expression in petals > in sepals > in stamens and carpels [23]. The expression of *CsAP2* gene in saffron is the highest in carpels, followed by perianth and stamen [24]. *PsAP2* gene is expressed the highest in carpels, followed by in petals of peony [18]. *AP2* was expressed in all four types of floral organs—sepals, petals, stamens, and carpels—and in developing ovules in *Arabidopsis* [4]. Ripoll et al. [25] reported that *AP2* acts to prevent overgrowth of the valve margin by repressing valve margin identity gene expression. In order to explore the relationship between expression level of the *APETALA2* gene and petal formation, we used qPCR and Western blot to investigate expression levels of *APETALA2* genes in red outer petals and yellow inner petals from four different developmental stages (flower bud stage, initial opening stage, full bloom stage, and decay stage) in the cultivar ‘ZiFengyu’. The results showed that the expression level of the *APETALA2* gene in the outer petals was significantly higher than that in the inner petals. Therefore, the expression level of the *APETALA2* gene was indeed associated with the petal formation of *P. lactiflora*. It is necessary to further verify the function of the *APETALA2* gene using tissue culture and transgenic technology.

## Supporting information

**Figure S1:** RACE cloning of *APETALA2* gene in *P. lactiflora*

**Figure S2:** cDNA Sequence information of *APETALA2* gene in *P. lactiflora*

**Supplemental Table:** 

**Supplemental Table:** 

**Supplemental Table:** 

**Supplemental Table:** 

**Supplemental Table:** 

**Supplemental Table:** 

## Abbreviations

ANS: anthocyanidin synthase
AP2: APETALA2
DEG: differentially expressed gene
DEP: differentially expressed protein
F3H: flavanone-3-hydroxylase
FBP7: floral binding protein 7
FBP11: floral binding protein 11
FLS: flavonol synthase
iTRAQ: isobaric tags for relative and absolute quantitation
PAL: phenylalanine ammonia-lyase
qRT-PCR: quantitative real-time polymerase chain reaction
RACE: rapid amplification of cDNA ends amplification
RNA-seq: RNA sequencing
