# Supplementary material for: Combination of transcriptome sequencing and iTRAQ proteome reveals the molecular mechanisms determining petal shape in herbaceous peony (Paeonia lactiflora Pall.)
Source: Biosci Rep. 2018 Dec 11;38(6):BSR20181485. doi: 10.1042/BSR20181485 (PMC6294620; doi:10.1042/BSR20181485)
Supplement: Supplementary file 1 [file bsr20181485_Supp1.pdf]

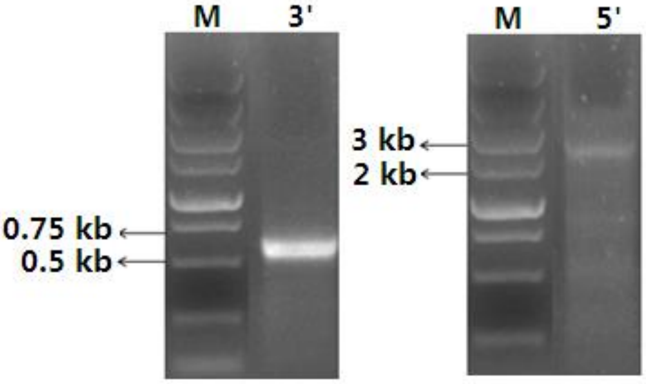

Figure S1 RACE cloning of *APETALA2* gene in *P. lactiflora*

Note: M: DL2000 marker; 3: 3' cDNA amplified fragment; 5: 5' cDNA amplified fragment

```
1       ACATGGGGATACTGGCCTCGTCTATGCTGTATTGGGCAGTTTGGGGGATTACAAGATCTT
61      GAAACATCTCCCCTTTCCTCCTTTTTGCAAACAAACTCACCTCGTAGTCATAGAGGAACG
121     AAGGTGCCAAGTCTCTAAAATTTTGATGGTGAGAGTGATGATCAGAAATCGGAGTCTCAG
181     AGAGTAATCAGCTTGATCAACAGTGTTGTGACGTAAAGAAAAATGTGGGATCTGAACGAT
                                                  M  W  D  L  N  D
241     TCACCTGATCGGAGGAGGGACCACGATGAATCCGATGACGACAGGGGTAAACGTGTTGAA
         S  P  D  R  R  R  D  H  D  E  S  D  D  D  R  G  K  R  V  E
301     TCAGTATCCAATGAGAATTCAAGTTCTTCAGTGGTGGTAATGGAAGATGGATCCGAGGAT
         S  V  S  N  E  N  S  S  S  S  V  V  V  M  E  D  G  S  E  D
361     GAAGAAGAAGGAGGAAGAGGCAGAACCAAGAAGCGGATCAGCAGCAAGATATTTGGGTTT
         E  E  E  G  G  R  G  R  T  K  K  R  I  S  S  K  I  F  G  F
421     TCTGTGACCCCAAATGAAGAAGAAGACTTGTCGTCGGAAAGTGACCCTCTTGTAACCCGG
         S  V  T  P  N  E  E  E  D  L  S  S  E  S  D  P  L  V  T  R
481     CAGTTCTTTCCGGTAGAGGGTTTAGAAATGGGGTCAGGGTCTGCTGGTGGTGGTGTATCT
         Q  F  F  P  V  E  G  L  E  M  G  S  G  S  A  G  G  G  V  S
541     GGGTTTCCAAGAGCTCATTGGGTCGGTGTAAAATTTTGTCAGTCGGAGCCTCTTGTACCC
         G  F  P  R  A  H  W  V  G  V  K  F  C  Q  S  E  P  L  V  P
601     GGAACCGGCAAGTCGGCGGAGGTTTCTCAGCCTTTGAAGAAAAGCCGGCGAGGTCCAAGG
         G  T  G  K  S  A  E  V  S  Q  P  L  K  K  S  R  R  G  P  R
661     TCTAGAAGCTCTCAATATCGTGGGGTTACCTTTTATCGTAGAACTGGCCGATGGGAGTCT
         S  R  S  S  Q  Y  R  G  V  T  F  Y  R  R  T  G  R  W  E  S
721     CATATATGGGATTGTGGAAAACAAGTTTATCTGGGTGGATTTGACACAGCACATGCAGCT
         H  I  W  D  C  G  K  Q  V  Y  L  G  G  F  D  T  A  H  A  A
781     GCCCGAGCATATGACAGAGCGGCCATCAAGTTCCGGGGAGTAGAGGCAGACATAAATTTC
         A  R  A  Y  D  R  A  A  I  K  F  R  G  V  E  A  D  I  N  F
841     AGTCTTGAAGATTACGAAGAAGACTTGAAACAGATGACTAATTTAACCAAAGAAGAATTT
         S  L  E  D  Y  E  E  D  L  K  Q  M  T  N  L  T  K  E  E  F
901     GTGCACGTACTTCGCCGACAGAGTACTGGGCTTCCTAGAGGTAGCTCCAAATATAGAGGT
         V  H  V  L  R  R  Q  S  T  G  L  P  R  G  S  S  K  Y  R  G
961     GTTACGTTGCATAAATGTGGAAGATGGGAAGCTCGAATGGGCCAATTTTTAGGCAAAAAG
         V  T  L  H  K  C  G  R  W  E  A  R  M  G  Q  F  L  G  K  K
1021    TATGTGTATTTGGGCCTTTTTGATACCGAAATTGAAGCTGCAAGGGCGTATGACAAAGCT
         Y  V  Y  L  G  L  F  D  T  E  I  E  A  A  R  A  Y  D  K  A
1081    GCAATTAAGTGCAATGGCAAGGACGCTGTCACCAACTTTGATCCCAGCATATATGAAAAT
         A  I  K  C  N  G  K  D  A  V  T  N  F  D  P  S  I  Y  E  N
1141    GAACTCAACTCCACTGAAGGTTCACGTAAATCTGGAGATCACAACCTTGATTTGAGCTTA
         E  L  N  S  T  E  G  S  R  K  S  G  D  H  N  L  D  L  S  L
1201    GGTAATTCCACCTCAAAGAGCAACAGTTCAGAATTCGGAGACAATAGTCAGTATATCACA
         G  N  S  T  S  K  S  N  S  S  E  F  G  D  N  S  Q  Y  I  T
1261    ATGAATAATGGGCATTCATTGCAGCCAATTCCAGCTGAAGCTGATTGGAGGAACAGTGGA
         M  N  N  G  H  S  L  Q  P  I  P  A  E  A  D  W  R  N  S  G
1321    TTTAGGCCCAAGCTAAACCCACATCAAGGGCAATGTACAAGTGAGGCAAACGAAACCATG
         F  R  P  K  L  N  P  H  Q  G  Q  C  T  S  E  A  N  E  T  M
1381    CAGCTTTTGGGCCAAACCCACTATCAAACTCCTAATGAAATGAACAGATATGGGCAGTTT
         Q  L  L  G  Q  T  H  Y  Q  T  P  N  E  M  N  R  Y  G  Q  F
1441    AGGAGACTGGGAGAAACCCAGATGCTTCACATTCTTCCATCTCAATTCAACTCATCAAAC
         R  R  L  G  E  T  Q  M  L  H  I  L  P  S  Q  F  N  S  S  N
1501    TATCAAGTGAGAATGAATCAGTTTCCAAGCAACAGCAACGGAAGCCGAATGATGGGAGTG
         Y  Q  V  R  M  N  Q  F  P  S  N  S  N  G  S  R  M  M  G  V
1561    AATGGAGGACGAGGAGGAGATCCTTGTCTGTACACTAGAGAGCAACAACAATGGCAGCAA
         N  G  G  R  G  G  D  P  C  L  Y  T  R  E  Q  Q  Q  W  Q  Q
1621    CCAGGTCCTCCTCAACTATTTGCAACTGCTGCAGCATCATCAGGATTCCCATCACAGATA
         P  G  P  P  Q  L  F  A  T  A  A  A  S  S  G  F  P  S  Q  I
1681    ATTACTCCCAGTAATACTAGGCCAAAAGAGAAGAGACATATAGCTATAATGGCGAGAGGG
         I  T  P  S  N  T  R  P  K  E  K  R  H  I  A  I  M  A  R  G
1741    ACTTATCAACTTAACATACGGCTTCCAAGGACAACTGTGGGGGAGGAGAGAGAAGGGTGA
         T  Y  Q  L  N  I  R  L  P  R  T  T  V  G  E  E  R  E  G  *
1801    CAGGGAAAGGGTTGAACCGGTTGTTGCAATGAGCACCCTTTCGAGTCTCCGAGGAAACGG
        TGTAGAGCGCTGTAAACGTTGGTTGAAAATTCCTATAAAATATTAAACGACATTACTATA
1921    TGGAAAAAAAAAAAA
```

Figure S2 cDNA Sequence information of *APETALA2* gene in *P. lactiflora*
